# Long-Term Survival among Octogenarians Undergoing Aortic Valve Replacement with or without Simultaneous Coronary Artery Bypass Grafting: A 22-Year Tertiary Single-Center Experience

**DOI:** 10.3390/jcm12144841

**Published:** 2023-07-22

**Authors:** Hannah Masraf, Davorin Sef, Sirr Ling Chin, Gabriel Hunduma, Vladimir Trkulja, Szabolcs Miskolczi, Theodore Velissaris, Suvitesh Luthra

**Affiliations:** 1 Wessex Cardiothoracic Centre, University Hospital Southampton, Southampton SO16 6YD, UK; hannah.masraf1@nhs.net (H.M.); slc1n20@soton.ac.uk (S.L.C.); gabriel.hunduma@uhs.nhs.uk (G.H.); szabolcs.miskolczi@uhs.nhs.uk (S.M.); theodore.velissaris@uhs.nhs.uk (T.V.); suvitesh.luthra@nhs.net (S.L.); 2 School of Medicine, University of Zagreb, 10000 Zagreb, Croatia; vladimir.trkulja@mef.hr

**Keywords:** aortic valve replacement, coronary artery bypass grafting, octogenarians, long-term survival

## Abstract

Background: The impact of concomitant coronary artery bypass grafting (CABG) on aortic valve replacement (AVR) in octogenarians is still debated. We analyzed the characteristics and long-term survival of octogenarians undergoing isolated AVR and AVR + CABG. Methods: All octogenarians who consecutively underwent AVR with or without concomitant CABG at our tertiary cardiac center between 2000 and 2022 were included. Patients with redo, emergent, or any other concomitant procedures were excluded. The primary endpoints were 30-day and long-term survival. The secondary endpoints were early postoperative outcomes and determinants of long-term survival. Univariable and multivariable logistic regression analyses were performed to identify independent predictors of 30-day mortality, and Cox regression analysis was performed for predictors of adverse long-term survival. Results: A total of 1011 patients who underwent AVR (83.0 [81.0–85.0] years, 42.0% males) and 1055 with AVR + CABG (83.0 [81.2–85.4] years, 66.1% males) were included in our study. Survival at 30 days and at 1, 3, and 5 years in the AVR group was 97.9%, 91.5%, 80.5%, and 66.2%, respectively, while in the AVR + CABG group it was 96.2%, 89.6%, 77.7%, and 64.7%, respectively. There was no significant difference in median postoperative survival between the AVR and AVR + CABG groups (7.1 years [IQR: 6.7–7.5] vs. 6.6 years [IQR: 6.3–7.2], respectively, *p* = 0.21). Significant predictors of adverse long-term survival in the AVR group included age (hazard ratio (HR): 1.09; 95% CI: 1.06–1.12, *p* < 0.001), previous MI (HR: 2.08; 95% CI: 1.32–3.28, *p* = 0.002), and chronic kidney disease (HR 2.07; 95% CI: 1.33–3.23, *p* = 0.001), while in the AVR + CABG group they included age (HR: 1.06; 95% CI: 1.04–1.10, *p* < 0.001) and diabetes mellitus (HR: 1.48; 95% CI: 1.15–1.89, *p* = 0.002). Concomitant CABG was not an independent risk factor for adverse long-term survival (HR: 0.89; 95% CI: 0.77–1.02, *p* = 0.09). Conclusions: The long-term survival of octogenarians who underwent AVR or AVR + CABG was similar and was not affected by adding concomitant CABG. However, octogenarians who underwent concomitant CABG with AVR had significantly higher in-hospital mortality. Each decision should be discussed within the heart team.

## 1. Introduction

Aortic valve stenosis (AS) and coronary artery disease (CAD) are the most common cardiac diseases in the aged population. The UK’s population is aging, with 3.2 million octogenarians (4.7%), resulting in the threefold greater proportion of octogenarians undergoing coronary artery bypass grafting (CABG) up to 7.2% over a 15-year period [1]. Despite the fact that the use of a transcatheter approach in octogenarians with AS has been increasing, the impact of concomitant CABG on aortic valve replacement (AVR) among octogenarian patients is still debated. In particular, this decision remains challenging among those octogenarians who are not high-risk for surgery (EuroSCORE II < 8%), who are unsuitable for transcatheter aortic valve implantation (TAVI), or who have heavily calcified CAD [2].

To the best of our knowledge, no randomized controlled trial focused on whether performing a concomitant CABG with AVR among octogenarians can be beneficial for their long-term survival is available. Recent studies have retrospectively compared at least 3-year follow-up periods between groups of patients who have received either isolated AVR or concomitant CABG and AVR. Although increased operative mortality of concomitant AVR and CABG has been reported, there appeared to be no additional mid-term survival risk compared to isolated AVR [3,4,5,6,7]. However, most of these reports are single-center and analyzed relatively small numbers of octogenarians. In a recent meta-analysis of 5382 octogenarian patients, Gallingani and colleagues found that late survival did not differ significantly between the two groups [8]. Furthermore, in another meta-analysis of retrospective observational studies comparing outcomes of patients undergoing AVR, D’Alessandro and colleagues found that AVR + CABG was associated with a significantly higher incidence of 30-day mortality, postoperative acute renal failure, need for dialysis, and prolonged mechanical ventilation as compared with AVR [9,10]. However, the reported results share limitations inherent to the retrospective design of the study and are limited by several unmeasured confounders, particularly since many of the octogenarians who underwent isolated AVR had their CAD treated percutaneously during the follow-up. Furthermore, data on predictors of adverse long-term survival among these two different groups of octogenarian patients are still lacking.

Based on these considerations, the primary aim of our study was to compare octogenarians who consecutively underwent AVR with or without CABG in our tertiary cardiac surgery center over the 22-year study period, and to evaluate the factors associated with long-term survival. As a secondary objective, early postoperative outcomes and survival were compared and investigated.

## 2. Materials and Methods

### 2.1. Study Design

We conducted a retrospective analysis of 2114 octogenarians who consecutively underwent AVR with or without concomitant CABG from June 2000 to April 2022 at the Department of Cardiac Surgery at University Hospital Southampton. Data were collected from the hospital’s databases, including the patient administration system (PAS) and the electronic clinical and management information system, (e-CAMIS, Yeadon, Leeds, UK). Data were analyzed as part of a quality improvement project registered with the Safeguard Team of University Hospital Southampton for the use of data in compliance with the local data protection policies (improvement project number 7376, 8 November 2022). No ethics committee approval was required, given that this study was retrospective and anonymized. All patients consented to the surgery and the use of their anonymized data for future studies. This work is reported in compliance with the STROCSS protocol [11]. Our preliminary data were presented at the Annual Meeting of the Society of Cardiothoracic Surgery in Great Britain & Ireland, Birmingham, UK, 19–21 March 2023.

The baseline demographics included data used for the NICOR (National Institute of Cardiac Outcomes Research, UK) database. Preoperative patients’ clinical data included demographics, clinical symptoms, comorbidities and cardiovascular risk factors (e.g., history of myocardial infarction, diabetes mellitus, hypertension, smoking history, chronic kidney disease), CAD, left main stem disease, left ventricular function, aortic valve pathology, and logistic EuroSCORE. Intraoperative and postoperative data included cardiopulmonary bypass (CPB) time, cross-clamp time (XCT), aortic prosthesis size, re-exploration for bleeding, new stroke, deep sternal wound infection (DSWI), in-hospital mortality, and survival during the follow-up. The primary endpoints were 30-day and long-term survival. The secondary endpoints were early postoperative outcomes (e.g., stroke, DSWI, and re-exploration for bleeding) and determinants of long-term survival. For the purposes of this study, octogenarian patients were categorized by procedure type (i.e., isolated AVR or AVR + CABG).

### 2.2. Patients

The inclusion criteria included all octogenarians who underwent AVR with or without concomitant CABG during the study period. Patients with redo, emergent, or any other concomitant procedures were excluded. The criteria for surgery were followed according to the guidelines of the American College of Cardiology/American Heart Association and European Society of Cardiology (ESC) and the European Association for Cardio-Thoracic Surgery (EACTS) at the time of surgery [2,12,13,14,15,16,17,18,19,20]. In the case of high-risk patients, each decision for management was reviewed by the institutional multidisciplinary team [17,18,21,22]. Coronary angiography was performed in all patients preoperatively to investigate the presence of associated CAD. A standard cardiac surgical approach was used through a complete median sternotomy. The left internal mammary artery was harvested in a pedicled fashion. The cannulation technique, myocardial protection, degree of hypothermia, valve implantation technique, and strategy for coronary revascularization were selected primarily based on the surgeon’s preference and their individual techniques. Long-term survival was determined from the PAS, e-CAMIS, and the electronic database of general practitioners’ medical records linked to the hospital database on the National Healthcare Service Spine Portal Summary Care Records (SCR) system.

### 2.3. Statistical Analysis

All clinical data were analyzed retrospectively through a review of prospectively collected electronic and archived medical records. Continuous variables are presented as medians ± interquartile ranges (IQRs). Categorical variables are presented as counts and percentages. Demographic and clinical preoperative and postoperative characteristics were compared between the AVR and AVR + CABG groups. Categorical data were compared using the chi-squared test or one-way ANOVA for variables with more than two groups, whereas continuous data were compared with Mood’s median test.

Univariable and multivariable logistic regression analyses were performed to identify independent predictors of 30-day mortality. Variables with a *p*-value < 0.20 were entered into a multivariable model. Cox regression survival analysis was performed to identify predictors of adverse long-term survival. Survival rates were calculated using the Kaplan–Meier method, and survival time was compared between groups using the log-rank test. A *p*-value < 0.05 was considered statistically significant. Statistical analyses were performed using Stata statistical software v17 (StataCorp. College Station, TX, USA, 2021).

## 3. Results

### 3.1. Preoperative Characteristics

The study population included a total of 2066 octogenarians who underwent either isolated AVR (1011 patients) or simultaneous AVR and CABG (1055 patients). The patients’ baseline demographics and preoperative clinical characteristics are presented in Table 1. The median age was 83.0 (IQR: 81.2–85.4) years, and the AVR + CABG group was predominantly male. As expected, the AVR + CABG group had a significantly higher proportion of three-vessel CAD (25.1% vs. 0.6%, *p* < 0.001), hypertension (71.8% vs. 61.7%, *p* < 0.001), smoking history (56.6% vs. 51.3%, *p* = 0.02), previous MI (13.9% vs. 2.8%, *p* < 0.001), and angina (59.0% vs. 37.1%, *p* < 0.001) as compared to the AVR group. The median logistic EuroSCORE was 10.0% in both groups (*p* = 0.34).

### 3.2. Perioperative Characteristics

Perioperative characteristics are presented in Table 2. As expected, both CPB (114 min vs. 79 min, *p* < 0.001) and XCT time (80 min vs. 58 min, *p* < 0.001) were significantly longer in the AVR + CABG group as compared to the AVR group. The 30-day mortality was significantly higher in the AVR + CABG group (3.8% vs. 2.1%, *p* = 0.02), while there was no significant difference in the stroke rate between the two groups (4.5% vs. 3.6%, *p* = 0.30). Postoperative survival is demonstrated in Table 3.

In a multivariable analysis, predictors of 30-day mortality after adjusting for confounders included age (odds ratio (OR): 1.13; 95% confidence interval (CI): 1.03–1.23, *p* = 0.01), chronic kidney disease (CKD) (OR: 4.73; 95% CI: 1.77–12.68, *p* = 0.002), CPB (OR: 1.02; 95% CI: 1.01–1.03, *p* < 0.001), and NYHA class ≥ 3 (OR: 1.87; 95% CI: 1.01–3.44, *p* = 0.05) (Table 4).

### 3.3. Long-Term Survival Analysis

**Figure 1:**
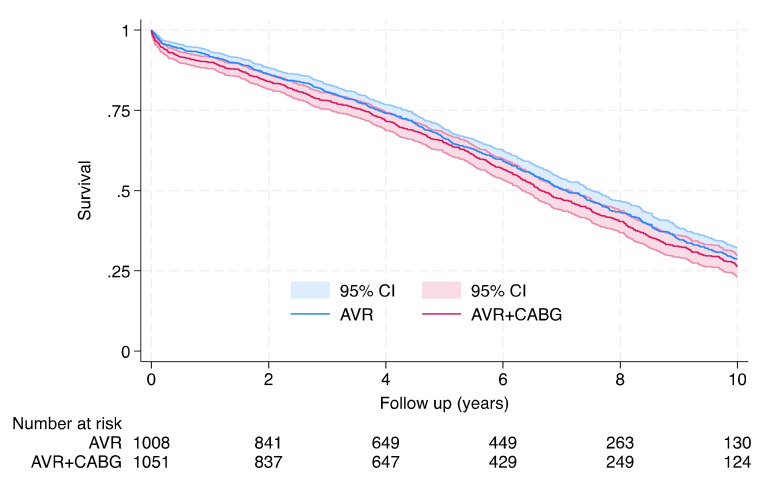
Kaplan–Meier estimate of overall survival for 1008 octogenarians who underwent AVR and 1051 who underwent AVR + CABG included in the analyses with 95% confidence intervals (CIs) (*p* = 0.21). The overall median was 6.9 years (95% CI: 6.6–7.2 years).

Survival for the entire group was estimated by Kaplan–Meier analysis (Figure 1). The median estimated postoperative survival was comparable between the AVR and AVR + CABG groups (7.1 years [IQR: 6.7–7.5] vs. 6.6 years [IQR: 6.3–7.2], respectively, *p* = 0.21) (Table 2). During long-term follow-up, the estimated postoperative survival at 1, 3, 5, and 7 years in the AVR group was 91.5%, 80.5%, 66.2%, and 50.3%, respectively, while in the AVR + CABG group it was 89.6%, 77.7%, 64.7%, and 47.2%, respectively (Table 3).

Significant predictors of adverse long-term survival among octogenarians who underwent AVR included age (hazard ratio (HR): 1.09; 95% CI: 1.06–1.12, *p* < 0.001), previous MI (HR: 2.08; 95% CI: 1.32–3.28, *p* = 0.002), CKD (HR 2.07; 95% CI: 1.33–3.23, *p* = 0.001), smoking history (HR: 1.24; 95% CI: 1.05–1.47, *p* = 0.01), and NYHA class ≥ 3 (HR: 1.18; 95% CI: 1.00–1.39, *p* = 0.05) (Table 5). Significant predictors of adverse long-term survival among octogenarians who underwent AVR + CABG included age (HR: 1.06; 95% CI: 1.04–1.10, *p* < 0.001) and diabetes mellitus (HR: 1.48; 95% CI: 1.15–1.89, *p* = 0.002) (Table 6). Type of surgery (AVR with concomitant CABG) was not an independent risk factor for adverse long-term survival (HR: 0.96; 95% CI: 0.79–1.16, *p* = 0.67) (Supplementary Table S1).

## 4. Discussion

One of the key challenges due to the increase in the elderly population remains determining when concomitant AVR and CABG should be performed in octogenarian patients rather than percutaneous intervention. This study provides a detailed benchmark of long-term survival following AVR with or without CABG among octogenarian patients over a 22-year period. The main finding of our study was that once past the initial in-hospital mortality, properly selected octogenarians have excellent long-term survival after AVR + CABG as compared to the isolated AVR group. We observed excellent median estimated postoperative survival among octogenarians, which was comparable between the AVR and AVR + CABG groups (7.1 years vs. 6.6 years, respectively, *p* = 0.21) despite the increased burden of advanced CAD in the AVR + CABG group. Furthermore, octogenarians who underwent concomitant CABG with AVR had significantly higher in-hospital mortality; therefore, each decision should be discussed within the heart team. Another important finding was that significant predictors of adverse long-term survival were male gender, age, previous MI, CKD, and NYHA class ≥ 3 in the AVR group, while age and diabetes mellitus were the significant predictors in the AVR + CABG group. Importantly, the type of surgery (AVR + CABG) was not an independent risk factor for adverse long-term survival.

Our findings are consistent with previous reports on AVR or AVR + CABG from high-volume centers among octogenarians [3,4,5,6]. However, due to their small-to-moderate sample sizes, most of these studies were inadequately powered to detect statistically significant differences. Therefore, Gallingani and colleagues recently performed a meta-analysis of retrospective observational studies that compared the survival of AVR and AVR + CABG in octogenarian patients and found that AVR + CABG was associated with a significantly higher incidence of mortality within the first year after surgery, while from 1- to 10-year follow-up no significant differences were observed between the two groups [8]. The authors concluded that it is useful to consider the favorable impact of CABG on the late survival of octogenarians who need treatment for AS associated with CAD [8]. Furthermore, Wang and colleagues reported a cohort of 197 octogenarians, with similar outcomes in both groups after the average follow-up of 4 years and estimated survival of 86.1% and 67.6% after 3 and 5 years in the AVR + CABG group, respectively, although they only analyzed factors associated with short-term survival [3]. Raja and colleagues reported comparable short-term outcomes for concomitant AVR and CABG among octogenarians compared to the isolated AVR group (9.6% vs. 7.4%, respectively, *p* = 0.35), justifying the performance of AVR + CABG in this high-risk group of carefully selected patients [23]. The Society of Thoracic Surgeons’ database report demonstrated an increased perioperative risk of mortality of 3.9% in the AVR group and 5.9% in the AVR + CABG subgroup of octogenarian patients, which was higher than reported in our study [24]. Interestingly, the authors observed a median postoperative survival of 5.9 years after AVR + CABG, which was somewhat lower than in our experience, and they concluded that the long-term prognosis after AVR with concomitant CABG is somewhat worse than after isolated AVR [24]. However, they acknowledged the absence of long-term follow-up for one-fourth of the STS elderly AVR cohort and underreporting of mortality in some subgroups as important limitations that might have resulted in an overestimate of the true long-term survival of patients [24]. Moreover, Ennker and colleagues reported a 30-day hospital mortality of 15.4% for octogenarians who underwent AVR + CABG [25]. Similarly, Kolh and colleagues reported a short-term mortality among octogenarian patients as high as 24% for AVR + CABG [26].

One of the main strengths of our study is that it provides evidence about factors associated with long-term survival, whereas several previous studies only investigated predictors of short-term mortality [3,23]. Importantly, concomitant CABG was not an independent risk factor for adverse long-term survival. A similar finding related to 30-day mortality was reported by Dell’Amore and colleagues, who showed that concomitant CABG was not an independent risk factor in octogenarians [4]. However, longer CPB time, which can be expected during concomitant AVR and CABG surgery, appeared to be associated with increased 30-day mortality after multivariable analysis (*p* < 0.001). This finding is similar to the previous report by Ennker and colleagues, who found that the difference in 30-day mortality was related to a higher risk of myocardial damage and arrhythmias resulting from a longer clamping and cardiopulmonary bypass time [25]. Furthermore, we demonstrated that in addition to age, several specific risk factors were associated with reduced long-term survival in the AVR group, such as a history of MI and NYHA class ≥ 3. This finding was expected, since patients with previous MI or more symptoms are “sicker” and fall into higher-risk subgroups of patients [27,28]. Similarly, patients with preoperative CKD will have worse long-term outcomes [29]. Another interesting finding is that the female survival advantage (HR: 0.82; 95% CI: 0.70–0.94, *p* = 0.006) over men still exists in octogenarians undergoing AVR with or without CABG (Supplementary Table S1). Importantly, this study expands on prior work by providing estimates of survival and determinants associated with adverse long-term survival among octogenarians that can be used directly for prognosis and to determine therapeutic options.

It can be also argued that it would be safer to use a percutaneous approach such as percutaneous coronary intervention (PCI) with TAVI in octogenarians because of its better safety profile. Over the last decade, the use of the transcatheter approach for octogenarian patients with AS has been increasing, even in intermediate- and low-risk patients, although patients with concomitant AS and CAD represent the most challenging group [30,31,32]. The prospective SURTAVI trial, which enrolled intermediate-risk patients with severe AS and non-complex CAD (SYNTAX score < 22), included 169 TAVI + PCI patients and 163 AVR + CABG patients [33]. No significant differences in the rates of all-cause mortality or disabling stroke at two years were found between the two groups (16%, vs. 14%, respectively, *p* = 0.69) [33]. However, as per the current guidelines, these results cannot be extrapolated to other patient groups with complex multivessel CAD including left main stem disease or diabetes mellitus [14]. Similarly, in a recent meta-analysis including three multicenter studies, no differences were found in 30-day mortality, stroke, or 2-year mortality between the TAVI + PCI and CABG + AVR groups [34]. However, rates of vascular complications, pacemaker implantation, and paravalvular regurgitation were consistently higher after TAVI [30,31,32,35,36]. Furthermore, patients with challenging or impossible transfemoral access, unsuitable aortic annular dimensions, low coronary ostia, or heavy leaflet calcifications and bicuspid aortic valves represent unfavorable characteristics for considering TAVI [2,37,38,39,40]. Although the PARTNER 1 trial found similar 30-day (3.4 and 6.5%, *p* = 0.07), and 5-year (67.8 and 62.4%) mortality rates between SAPIEN TAVI and AVR among high-risk patients with aortic stenosis, the combined stroke and transient ischemic attack rate was more frequent in the patients assigned to TAVI at 30 days (5.5% TAVI vs. 2.4% surgery, *p* = 0.04) and at 1 year (8.7% TAVI vs. 4.3% surgery, *p* = 0.04) [35]. Moderate or severe aortic regurgitation occurred in 40 (14%) out of 280 patients in the TAVI group and 2 (1%) out of 228 in the AVR group (*p* < 0.0001) and was associated with an increased 5-year risk of mortality in the TAVI group [35]. Lastly, in comparison with TAVI, the surgical approach allows for careful debridement and complete removal of the calcified aortic valve, with appropriate sizing of the prosthetic valve and root enlargement if required [2]. However, more robust data with further prospective randomized comparisons between these treatment arms would be required to have clear evidence regarding the best method.

Limitations are inherent to the retrospective observational study design and its restriction to a single institution. However, we used a prospectively maintained hospital database managed by several database managers who periodically check the accuracy of data entry, and we included all consecutive patients over the long study period. Another strength of our study is that since long-term survival was determined from PAS, e-CAMIS, and the electronic database of general practitioners’ medical records linked to our hospital database, this provided accurate mortality data. Furthermore, there was a potential selection bias, since each treatment decision in this challenging group of patients was reviewed either individually or in our multidisciplinary team meetings, and the best treatment was proposed for each patient based on a benefit–risk balance. Overall, octogenarians who were considered for more complex surgeries, such as AVR + CABG, were “healthier” and with fewer comorbidities. Additionally, we could not account for non-cardiac comorbidities or other treatments as potentially relevant baseline or post-baseline confounders. This study has another limitation of being retrospective and nonrandomized, and it is subject to bias related to potential subsequent PCI procedures in both groups of patients. Lastly, we did not have a comparison group with octogenarians who underwent either minimally invasive approaches or TAVI with or without PCI [22,41,42]. However, this would require conducting further prospective and multicenter studies.

## 5. Conclusions

Long-term survival following AVR + CABG in octogenarian patients was satisfactory and was similar to that of octogenarians undergoing isolated AVR after a median follow-up of 7 years. However, octogenarians who underwent concomitant CABG with AVR had significantly higher 30-day mortality. We also found that there were no significant differences in early postoperative complications (e.g., stroke, re-exploration, and DSWI) between the two groups. Therefore, each decision should be discussed within the heart team. Further prospective and large multicenter studies are needed to compare the long-term outcomes with those of octogenarians who undergo minimally invasive or percutaneous approaches.

## Figures and Tables

**Table 1:** Preoperative characteristics.

Characteristics	Total (n = 2066)	AVR (n = 1011)	AVR+CABG (n = 1055)	*p*-Value
Age (years)	83.0 (81.0–85.0)	83.0 (81.0–85.0)	83.0 (81.2–85.4)	0.11
Gender (male)	1122 (54.3)	425 (42.0)	697 (66.1)	**<0.001**
BMI (kg/m^2^)	26.2 (23.5–29.4)	26.2 (23.4–29.4)	26.3 (23.7–29.4)	0.43
Angina	997 (48.3)	375 (37.1)	622 (59.0)	**<0.001**
NYHA class ≥ 3	820 (39.7)	427 (42.2)	393 (37.3)	**0.02**
Previous MI	175 (8.5)	28 (2.8)	147 (13.9)	**<0.001**
Diabetes mellitus	206 (10.0)	92 (9.1)	114 (10.8)	0.20
Hypertension	1381 (66.8)	624 (61.7)	757 (71.8)	**<0.001**
Smoking history	1116 (54.0)	519 (51.3)	597 (56.6)	**0.02**
CKD	54 (2.6)	26 (2.6)	28 (2.7)	0.91
Three-vessel CAD	271 (13.1)	6 (0.6)	265 (25.1)	**<0.001**
LMS	n/a	n/a	142 (14.9)	**-**
LVEF ≤ 30 *	68 (4.4)	38 (3.8)	30 (5.6)	0.10
*AV pathology*				
Stenosis	1594 (77.2)	761 (75.3)	833 (79.0)	**0.05**
Regurgitation	126 (6.1)	81 (8.0)	45 (4.3)	<0.001
Mixed	346 (16.7)	169 (16.7)	177 (16.8)	0.97
Log EuroSCORE (%)	10.1 (7.5–16.5)	10.0 (7.7–16.9)	10.0% (7.0–16.0)	0.34

Data are presented as n (%) or median (quartiles). Abbreviations: AVR, aortic valve replacement; BMI, body mass index; CABG, coronary artery bypass grafting; CAD, coronary artery disease; CKD, chronic kidney disease; LMS, left main stem disease; LVEF, left ventricular ejection fraction; MI, myocardial infarction; n/a, not applicable; NYHA, New York Heart Association; * Data are presented as valid percentages when considering missing data.

**Table 2:** Intraoperative and postoperative characteristics.

Characteristics	Total (n = 2066)	AVR (n = 1011)	AVR+CABG (n = 1055)	*p*-Value
CPB (min)	97.0 (75–126)	79 (65–102)	114 (93–138)	**<0.001**
XCT (min)	70 (54–90)	58 (48–76)	80 (65–99)	**<0.001**
*Aortic prosthesis size* *				
<21 mm	536 (25.9)	463 (45.8)	73 (6.9)	**<0.001**
21 mm	648 (31.4)	359 (35.5)	289 (27.4)	**<0.001**
23 mm	770 (37.3)	364 (36.0)	406 (38.5)	0.24
25 mm	380 (18.4)	141 (13.9)	239 (22.7)	**<0.001**
27 mm	71 (3.4)	32 (3.2)	39 (3.7)	0.51
>27 mm	11 (0.5)	6 (0.6)	5 (0.5)	0.71
*Early complications*				
Re-exploration for bleeding	54 (2.6)	25 (2.5)	29 (2.7)	0.69
DSWI	2 (0.1)	0 (0)	2 (0.2)	0.17
Stroke	83 (4.0)	36 (3.6)	47 (4.5)	0.30
30-day mortality	61 (3.0)	21 (2.1)	40 (3.8)	**0.02**
Median survival, years	6.9 (6.6–7.2)	7.1 (6.7–7.5)	6.6 (6.3–7.2)	0.21

Data are presented as n (%) or median (quartiles). Abbreviations: AVR, aortic valve replacement; CABG, coronary artery bypass grafting; CPB, cardiopulmonary bypass time; DSWI, deep sternal wound infection; XCT, cross-clamp time; * Data are presented as valid percentages when considering missing data.

**Table 3:** Postoperative survival during long-term follow-up.

Interval Time	AVR Survival (SE)	AVR+CABG Survival (SE)	Overall Survival (SE)	*p*-Value
1 year	91.5% (0.9%)	89.6% (0.9%)	90.5% (0.7%)	0.12
3 years	80.5% (1.3%)	77.7% (1.3%)	79.1% (1.0%)	0.14
5 years	66.2% (1.6%)	64.7% (1.5%)	65.4% (1.1%)	0.56
7 years	50.3% (1.7%)	47.2% (1.7%)	48.8% (1.2%)	0.57
10 years	28.8% (1.8%)	26.4% (1.7%)	27.6% (1.3%)	0.24

Abbreviations: AVR, aortic valve replacement; CABG, coronary artery bypass grafting; SE, standard error.

**Table 4:** Univariable logistic regression model for predictors of 30-day mortality (<0.2 entered into the multivariable model).

Variable	OR (Univariable)	95% CI	*p*-Value	OR (Multivariable)	95% CI	*p*-Value
Type of operation (AVR+CABG)	1.86	1.09–3.17	**0.02**	0.71	0.29–1.75	0.46
Age	1.08	0.99–1.18	0.08	1.13	1.03–1.23	**0.01**
Gender (female)	0.62	0.36–1.05	0.08	0.82	0.45–1.50	0.52
BMI (kg/m^2^)	0.95	0.90–1.01	0.11	0.96	0.89–1.02	0.19
Angina	1.68	1.00–2.83	0.05	1.24	0.60–2.22	0.48
NYHA class ≥ 3	1.70	1.01–2.87	0.05	1.87	1.01–3.44	0.05
Previous MI	2.13	1.06–4.27	**0.03**	0.98	0.41–2.34	0.97
Diabetes mellitus	1.61	0.80–3.21	0.18	1.09	0.46–2.57	0.86
Hypertension	1.14	0.96–1.34	0.14	1.13	0.93–1.36	0.21
Smoking history	1.36	0.80–2.29	0.26			
CKD	4.45	1.83–10.83	**0.001**	4.73	1.77–12.68	**0.002**
CAD	2.13	1.16–3.89	**0.01**	1.87	0.73–4.81	0.19
CPB	1.01	1.01–1.02	**<0.001**	1.02	1.01–1.03	**<0.001**
XCT	1.02	1.01–1.02	**<0.001**	0.99	0.97–1.00	0.13
Aortic bioprosthesis size	0.91	0.70–1.18	0.48			
Log EuroSCORE	1.04	0.90–1.20	0.60			

Abbreviations: AVR, aortic valve replacement; BMI, body mass index; CABG, coronary artery bypass grafting; CAD, coronary artery disease; CKD, chronic kidney disease; CI, confidence interval; CPB, cardiopulmonary bypass time; MI, myocardial infarction; NYHA, New York Heart Association; OR, odds ratio; XCT, cross-clamp time.

**Table 5:** Predictors of adverse long-term survival (Cox regression survival analysis) among octogenarians who underwent aortic valve replacement.

Variable	HR	95% CI	*p*-Value
Gender (female)	0.84	0.68–1.03	0.09
Age	1.09	1.06–1.12	**<0.001**
Previous MI	2.08	1.32–3.28	**0.002**
Diabetes mellitus	0.90	0.68–1.31	0.49
Angina	1.02	0.86–1.20	0.85
NYHA class ≥ 3	1.18	1.00–1.39	0.05
Smoking history	1.24	1.05–1.47	**0.01**
Hypertension	0.99	0.94–1.17	0.87
CKD	2.07	1.33–3.23	**0.001**
Three-vessel CAD	1.00	0.82–1.22	0.99
LVEF	1.06	0.92–1.23	0.39
Aortic prosthesis size	0.93	0.85–1.03	0.15
Logistic EuroSCORE	1.06	1.00–1.12	**0.04**

Abbreviations: CAD, coronary artery disease; CKD, chronic kidney disease; CI, confidence interval; LVEF, left ventricular ejection fraction; MI, myocardial infarction; NYHA, New York Heart Association; HR, hazard ratio.

**Table 6:** Predictors of adverse long-term survival (Cox regression survival analysis) among octogenarians who underwent AVR + CABG.

Variable	HR	95% CI	*p*-Value
Gender (female)	0.82	0.65–1.03	0.09
Age	1.06	1.04–1.10	**<0.001**
Previous MI	1.23	0.99–1.52	0.06
Diabetes mellitus	1.48	1.15–1.89	**0.002**
Angina	0.99	0.83–1.17	0.87
NYHA class ≥ 3	1.09	0.86–1.38	0.50
Smoking history	0.94	0.79–1.13	0.52
Hypertension	1.01	0.84–1.21	0.94
CKD	1.44	0.93–2.23	0.10
CAD	1.04	0.93–1.16	0.52
Aortic prosthesis size	1.05	0.94–1.16	0.38
Logistic EuroSCORE	0.98	0.85–1.13	0.77
Number of grafts	0.91	0.83–1.01	0.07
LMS stenosis	0.90	0.71–1.15	0.41

Abbreviations: CAD, coronary artery disease; CKD, chronic kidney disease; CI, confidence interval; LMS, left main stem disease; MI, myocardial infarction; NYHA, New York Heart Association; HR, hazard ratio.

## Data Availability

Data can be made available upon reasonable request to the corresponding author, due to privacy/ethical restrictions.

## Supplementary Materials

The following supporting information can be downloaded at: https://www.mdpi.com/article/10.3390/jcm12144841/s1, Table S1: Predictors of adverse long-term survival (Cox regression survival analysis) of all octogenarians who underwent AVR with or without CABG. Table S2: The full revised STROCSS 2021 checklist.
